# Acceleration for Gifted Students in Inclusive Education: Finnish and Swedish Physics and Chemistry Teachers’ Experience

**DOI:** 10.3390/bs16081329

**Published:** 2026-08-03

**Authors:** Taina Makkonen, Valerie Margrain

**Affiliations:** Department of Educational Studies, Karlstad University, SE-651 88 Karlstad, Sweden; valerie.margrain@kau.se

**Keywords:** acceleration, gifted education, inclusive education, science teachers

## Abstract

This exploratory survey study examines the use of accelerative practices among lower secondary physics and chemistry teachers in Finland and Sweden (*N* = 160), where a longstanding emphasis on educational equality and inclusion has limited attention to the needs of gifted students. Although international research demonstrates strong academic and socio-emotional benefits of acceleration for gifted students, evidence suggests that Nordic schools rarely implement such practices systematically. The present study investigates teachers’ experiences with four accelerative approaches relevant to the Nordic context—early entrance, grade skipping, subject-based acceleration, and curriculum compacting—and analyses how these practices shape instructional decision-making in science classrooms. Using a mixed-methods design combining descriptive and inferential statistics with inductive content analysis, the study reveals that acceleration is infrequently used in both countries, though Swedish teachers report higher implementation rates than Finnish teachers. Teachers with prior knowledge of gifted education were more likely to employ accelerative practices across all categories. Teachers reported limited time and material resources as barriers to implementation. The findings illustrate a persistent gap between policy-permitted accelerative opportunities and actual inclusive science education classroom practice, highlighting that meeting the learning needs of gifted students requires strengthened teacher education, policy support, and resource allocation.

## 1. Introduction

This exploratory survey study examines the use of acceleration practices among physics and chemistry teachers (*N* = 160) in inclusive lower secondary education in two Nordic countries, Finland and Sweden. Acceleration can be defined as a set of strategies for addressing the needs of students who require more challenge than a regular age- or grade-level-based curriculum can provide (Brody, 2004). A particular need for acceleration exists in heterogeneous classrooms, where gifted students may spend much of their time practising skills they have already learnt, reviewing content they have already mastered, or using insufficiently challenging learning materials (A. Robinson et al., 2007). Previous research has firmly established that acceleration is highly effective in terms of gifted students’ academic achievement (Assouline et al., 2015; Hattie, 2009; Rogers, 2015; Steenbergen-Hu et al., 2016). Several studies have also provided evidence of positive or neutral psychological (Bernstein et al., 2021; Ma, 2002; Rogers, 2015; see also Neihart, 2007; Wiley, 2016) and social (Gross, 2006; Lee et al., 2012; Rogers, 2015; see also Cross et al., 2015; Neihart, 2007) effects associated with certain forms of acceleration. Although a variety of accelerative options are available in the education systems of Finland and Sweden, there is no evidence that they are implemented frequently or systematically (Makkonen et al., in press), and when they are used, they may be limited to only a few subjects (Westling Allodi & Szabo, in press). Overall, little is known about the actual use of acceleration in these two countries (Ivarsson, 2024; Laine et al., 2025). Focusing on acceleration practices, rather than on other gifted education strategies, is particularly important given the well-documented effectiveness of acceleration. For example, meta-analyses have reported significantly larger effect sizes for acceleration than for enrichment (Hattie, 2009). In addition, some research has examined other practices relevant to gifted students in inclusive education, such as differentiated instruction (Ardenlid, 2025; Ardenlid et al., 2025). However, findings from systematic reviews of differentiated instruction are mixed and often indicate only small effects on academic and affective-motivational outcomes (e.g., Barbier et al., 2023; Ziernwald et al., 2022).

There is no formal national definition of giftedness in either Finland or Sweden, and the national educational agencies do not provide a specific, operational definition. Instead, high ability is situated within broader frameworks of inclusive and equitable education (Finnish National Agency for Education, 2025; Swedish National Agency for Education, 2025). Within Nordic comprehensive education systems that prioritise equity and inclusion, the lack of formal gifted education policy does not necessarily imply the absence of provision; rather, it suggests that support for high-ability learners is embedded within broader differentiated instruction and inclusive pedagogical practices (Tirri & Kuusisto, 2013). Although this study focuses explicitly on Finland and Sweden, the findings can be of interest to other contexts in which there are no policies about gifted education and acceleration.

In the absence of explicit national policies on gifted education in Finland and Sweden, a theoretical positioning can nevertheless be grounded in Gagné’s (2004, 2021) Differentiating Model of Giftedness and Talent (DMGT), which distinguishes between innate abilities (giftedness) and systematically developed skills (talent). The DMGT emphasises the crucial mediating role of environmental catalysts such as schooling (Gagné, 2004, 2021), which is particularly useful in Nordic contexts where formal identification is rare and regular schools act as the primary context for supporting talent development. From a DMGT perspective, Finnish and Swedish schools can be understood as key developmental contexts where teachers, curriculum flexibility, and sociocultural values act as catalysts that may either facilitate or constrain the transformation of natural abilities into high-level competencies. However, without explicit recognition or targeted structures for gifted learners, there is a risk that the needs of these students remain implicit or under-addressed, relying heavily on teacher awareness and local practices rather than systemic support (Persson, 2010). Thus, Gagné’s framework helps to theorise how, even in policy-silent contexts, giftedness exists as a natural endowment while schools play a critical, though uneven, role in nurturing talent development through inclusive yet potentially diffuse educational approaches.

A notable incongruence prevails in both Finland and Sweden between accelerative opportunities and educators’ attitudes towards them. Options such as grade skipping and subject-based acceleration are supported by law (Laine et al., 2025; Liljedahl, 2017). However, a Finnish study found that primary-school teachers’ attitudes towards acceleration were negative, although their attitudes towards gifted education were generally positive and supportive of differentiated instruction (Laine et al., 2019). A study of Swedish principals reported that, from the principals’ perspective, grade skipping is problematic and should be avoided, and that acceleration should mainly be conducted in age-appropriate classes (Ivarsson, 2024). Educators’ negative attitudes towards acceleration have also been reported in other countries, such as Ireland (Cross et al., 2018), the Netherlands (Smeets et al., 2023), New Zealand (Wardman, 2017), South Korea (Woo et al., 2022), Türkiye (Kunt & Tortop, 2017), and the United States (US) (Lupkowski-Shoplik et al., 2015). However, it may be assumed that resistance to acceleration in Finland and Sweden is even more persistent—an assumption grounded in the Nordic countries’ overall educational and societal value base, characterised by a high level of egalitarianism, equity, and inclusion (Persson, 2010; Tirri & Margrain, in press).

Gifted education is a highly context-dependent field of research (Frantz & McClarty, 2016; Tourón & Freeman, 2018). A definition for giftedness by Gagné (2004) states: ‘Giftedness designates the possession and use of untrained and spontaneously expressed natural abilities (called outstanding aptitudes or gifts), in at least one ability domain, to a degree that places an individual at least among the top 10 per cent of age peers’ (p. 120). However, without recognition or support, many gifted individuals may not develop their innate potential. Results in gifted education research obtained in other countries cannot be directly applied to Finland and Sweden, where the ‘one school for all’ approach is deeply embedded. In such a context, giftedness is considered taboo (Ivarsson, 2024; Tirri, 2022), elitist (Mattsson & Bengmark, 2011; Svedin, 2023), and invisible or suppressed (Sims, 2023). Instead, while egalitarianism, uniformity, and inclusion are emphasised, individuals may feel uncomfortable ‘standing out’, and it is easier to use the term talent development than giftedness (Tirri, 2022). Furthermore, gifted education policies are non-existent or emerging (Tirri & Margrain, in press), gifted students’ needs are neglected in schools’ instructional practices (Tirri & Kuusisto, 2013), and home–school collaboration to support gifted students is limited (Ekesryd Nordström, 2025). On the other hand, recent critique indicates that the ‘one school for all’ has not achieved its ambition that all children’s needs should be able to be met in the regular classroom. This attention to unmet learning needs has mostly focused on students who are developmentally behind; however, the discussion potentially creates a space in which gifted education can be recognised alongside wider inclusive education implementation (Barow & Berhanu, 2021; Magnússon, 2020). Across all Nordic countries, there has been increasing awareness of gifted education in recent years—in policy, research, parent organisations, and the media (Tirri & Margrain, in press).

Comparing acceleration practices in Finland and Sweden is worthwhile, given their shared emphasis on inclusive education and egalitarianism, as well as generally negative attitudes towards giftedness. However, there are also notable differences, such as students’ achievement levels in science on the Programme for International Student Assessment (PISA), which are higher in Finland, and the level of diversity in the student population, which is higher in Sweden (Organisation for Economic Co-Operation and Development, 2023). There is also a considerably higher proportion of independently operated schools in Sweden than in Finland (Swedish Institute, 2026; Tirri & Kuusisto, 2013). Additionally, the social status of the teaching profession is generally higher in Finland than in Sweden (Organisation for Economic Co-Operation and Development, 2025). Furthermore, in Sweden, a government recommendation (not yet implemented at the time of writing) instructed the Swedish National Agency for Education to develop a national strategy and action plan for gifted students, including acceleration opportunities (Rantsi, 2024). In Finland, no such plan has been introduced.

Inclusive education and acceleration are often seen as competing priorities, yet research suggests they can be complementary when inclusion is understood as providing appropriate challenge for all learners (Dare & Nowicki, 2018). More specifically, an inclusive approach ensures equity by providing each learner, including gifted learners, with the educational support and opportunities they need (Baccassino & Pinnelli, 2023). Acceleration—such as grade skipping or subject-based advancement—has consistently been shown to produce positive academic outcomes for gifted students without negative social-emotional effects (Steenbergen-Hu & Moon, 2011). From an inclusive education perspective, meeting learners’ diverse needs includes ensuring that highly able students are not constrained by age-based norms but are instead given access to appropriately challenging curricula (Tomlinson, 2014). In this sense, acceleration can be viewed as a form of differentiated instruction that aligns with inclusive principles by enabling equity of opportunity rather than uniform provision (Dare & Nowicki, 2019, 2023). Thus, rather than contradicting inclusion, acceleration supports it by recognising that equity involves responsiveness to both learning difficulties and advanced capabilities.

Physics and chemistry were chosen as the specific disciplinary focus of the study, as high-level abilities in science are recognised as playing a critical role in promoting innovation to address current and future global challenges (United Nations, 2019). Fostering the talent development of high-ability students in science can help solve real-world problems in ways that significantly benefit society (Wai & Lovett, 2021). In addition, gifted students have the same legal and moral right as their peers to educational experiences that are appropriately challenging and responsive to their learning needs; consequently, teachers have an ethical responsibility to ensure equitable educational opportunities for all students (Tirri & Laine, 2017). It is thus important to understand how educators support the talent development of scientifically gifted students in inclusive school systems, where education predominantly occurs in mixed-ability classrooms and where no special gifted programmes, tracking systems, or streamed education are used.

A survey questionnaire examining teachers’ use and experiences of acceleration was designed as part of a larger research project. Data from 160 physics and chemistry teachers were analysed using statistical methods and inductive content analysis. The objective was to produce exploratory survey evidence on a topic not previously covered in Nordic gifted education literature; we will continue the research by interviewing teachers, students, and parents and by including lesson plans for data triangulation. The research questions guiding the present study were as follows: How do physics and chemistry teachers in Finland and Sweden use acceleration to meet the needs of gifted students? How do these practices vary according to teacher characteristics, such as country (Finland vs. Sweden), gender, knowledge of gifted education, and years of teaching experience?

### 1.1. Acceleration

Acceleration was defined concisely about 80 years ago as ‘progress through an educational program at rates faster or ages younger than conventional’ (Pressey, 1949, p. 2). However, as VanTassel-Baska (1992/2004) points out, acceleration may be mistakenly perceived solely as an intervention to speed up an individual’s education, when it should more accurately refer to ‘the rapid rate of a child’s cognitive development, not the educational intervention provided’ (p. 70). She further emphasises the goal of such interventions, namely matching services and curriculum to a gifted individual’s readiness and needs. That said, the focus of this study is not on developmental perspectives per se (e.g., learning readiness, ability, or talent development as a cognitive process). Instead, we examine acceleration from an educational—more specifically, pedagogical—perspective to provide new insights into instructional practices that have been infrequently investigated in the Nordic inclusive context. Moreover, the authors’ background in educational sciences, rather than psychology, further justifies this focus.

Southern and Jones (2015) list 20 different acceleration practices, which can be further divided into two broader categories: subject-based and grade-based acceleration (Assouline et al., 2021; Lupkowski-Shoplik et al., 2015; Rogers, 2015). Subject-based acceleration, also called content-based acceleration, focuses on a student’s specific area of demonstrated ability and may occur within their regular class or, on a part-time basis, in another learning environment (Riegel & Behrens, 2022). Examples of subject-based acceleration are single-subject acceleration and curriculum compacting (Lupkowski-Shoplik et al., 2015); these will be described in more detail below. In turn, grade-based acceleration reduces the time the student spends on their entire schooling, and it is thus the most visible form of acceleration (Southern & Jones, 2004). Examples of grade-based acceleration include whole-grade acceleration—typically referring to grade skipping—and early entrance to formal schooling (Lupkowski-Shoplik et al., 2015).

Not all 20 forms of acceleration are central to every education system. Laine et al. (2025) list four acceleration opportunities that are conceptually relevant to the Nordic basic education context: curriculum compacting, early entrance, grade skipping, and subject-based acceleration. These opportunities are permitted by legislation and are used in at least some schools in both Finland and Sweden; they are also presented in one of the few books on gifted education published in Finland to date (Laine et al., 2025). The results of this study report Finnish and Swedish teachers’ experiences with these four acceleration approaches.

Curriculum compacting is an approach in which curricular adjustments are made for a gifted student at any grade level in any subject or curricular area (Reis et al., 1998/2016). It begins with pre-assessing a student’s level of knowledge and skills, followed by working on content that is not yet mastered, at a rate and a level appropriate to that student (A. Robinson et al., 2007). The time saved is used for enrichment activities, such as projects, or for studying advanced-level content (A. Robinson et al., 2007). For example, teachers can provide more difficult texts, incorporate real-world content, teach models or graphic organisers for thinking and problem-solving, and add creative components, such as projects that combine advanced learning with synthesising information and creating artefacts (VanTassel-Baska & Stambaugh, 2005). However, as Laine et al. (2025) point out, curriculum compacting does not mean that students are left on their own or given additional tasks of the same (basic) level; they require not only appropriate challenges but also consistent teacher support.

In the study by Reis et al. (1998/2016) among high-ability elementary school students (*N* = 336) in the US, no differences in achievement were found between students whose curriculum was compacted and those who received regular instruction. The findings were similar across mathematics, science, social sciences, and reading. It was also found that even when 40–50% of content was eliminated, the high scores were maintained among students who had a very high level of ability and advanced subject knowledge prior to instruction. There was no decline in students’ scores even when the added or adjusted material was outside the compacted content area. In Rogers’s (2015) research synthesis—covering meta-analyses, best-evidence syntheses, and other research published in 1990–2013—the mean effect sizes for curriculum compacting were small but positive with regard to academic and psychological outcomes.

The second practice theoretically relevant to the Nordic context is starting school a year earlier than the standard age (Laine et al., 2025). This option gives learners access to a more challenging learning environment, enables them to develop persistence, and prevents boredom and underachievement (Colangelo et al., 2004). It also enables learners to integrate directly into a peer group (N. M. Robinson, 2004). From an academic and social perspective, early entrance may be an easier alternative than, for example, grade skipping in later years of schooling (Lupkowski-Shoplik et al., 2015).

Group comparisons by Gagné and Gagnier (2004) found no substantial differences between early entrants to kindergarten (*N* = 98) and their regularly admitted peers regarding social integration, maturity towards school tasks, or behaviour. In grade 2, early entrants’ academic achievement was higher than that of regularly admitted peers. However, in the qualitative part of the study, teachers reported adjustment issues in a relatively large proportion of early entrants; this may reflect educators’ negative attitudes towards early entrance (Gagné & Gagnier, 2004). Lupkowski-Shoplik et al. (2015) emphasise differentiating between two types of early-entrance research: studies that compare young but unselected students (e.g., those with birthdays early in the year) with regular-age students often show immaturity issues among early entrants, whereas studies involving carefully selected high-ability students show more positive effects. Overall, Rogers’s (2015) research synthesis found a moderate positive academic effect, a small positive social adjustment effect, and a small negative psychological adjustment effect of early entrance; the synthesis covered early entrance to first grade or kindergarten.

Grade skipping, the third option applicable in the context of this study, concerns all subjects at the same time and interferes with an established peer group. While the academic benefits of grade skipping are well established (Rogers, 2015), findings related to its social-emotional aspects are mixed. Hoogeveen et al. (2009) compared the peer-group social status, self-concept, and behaviour reputations of 53 grade-accelerated Dutch secondary school students and their 304 nonaccelerated (regular) grade peers. Grade-accelerated students had lower social status but a higher school-related self-concept than their peers. In turn, social self-concept was less positive among accelerated students than it was among their grade peers; however, for girls the difference disappeared within two years. Regarding behaviour reputation, conceit was associated more frequently with the accelerated students, whereas the grade peers were considered to be more cooperative, humorous, helpful, social, and leading. In a later study, the researchers examined the self-concept and social contacts of gifted accelerated students (*n* = 148) and gifted nonaccelerated students (*n* = 55) across a wider age range (Hoogeveen et al., 2012); the accelerated students had skipped grade(s), entered school early, or progressed through grades more quickly than usual. Only minimal differences in social-emotional characteristics were found between these groups, with the few differences favouring accelerated students. Wardman (2017) reported positive New Zealand perceptions of acceleration via full-year grade skipping, across different data collections. Her survey study of 455 high school teachers showed predominantly positive perceptions, and a smaller retrospective study of 12 accelerated students reported positive experiences from multiple viewpoints: the former students, their parents, and school administrators. In line with these results, findings from the research synthesis by Rogers (2015) show moderate positive social and psychological adjustment effects for grade skipping, in addition to a strong positive academic effect. Nevertheless, contrasting evidence regarding psychosocial outcomes also exists. For example, Kretschmann et al. (2016) found persistently weaker peer relations among grade-accelerated elementary school students (*n* = 96) compared to their non-accelerated peers. They also observed motivational disadvantages among grade-accelerated girls.

The fourth option—subject-based acceleration—offers a lighter alternative to grade skipping (Laine et al., 2025). The concept has been used in the literature as an umbrella term referring to various subject-related approaches (e.g., Lupkowski-Shoplik et al., 2015; Riegel & Behrens, 2022) that allow flexibility in progressing through curriculum or expose students to knowledge and skills beyond expected grade levels (Rogers, 2015). One example is single-subject acceleration, in which a student is accelerated in one subject but progresses at the regular pace in others (Rogers, 2015). Southern and Jones (2015) list synonymous terms such as subject-matter acceleration, partial acceleration, and content-based acceleration; in these approaches, the student can be placed in a higher-grade-level class for part of the day in one or more subjects. Alternatively, the student works with materials from higher grade levels while remaining in their regular class. Subject acceleration may also be implemented beyond school hours and in out-of-school settings (Southern & Jones, 2015).

In a longitudinal study of secondary school students accelerated in mathematics, Ma (2002) observed that subject acceleration predicted a long-term increase in gifted students’ (*N* = 276) self-esteem. In a qualitative study by Lee et al. (2010), the researchers interviewed 17 minority students in grades 6–9 who had been accelerated in mathematics, as well as seven mathematics or science teachers. The students’ experiences of subject acceleration were positive in both academic and social-emotional respects. The teachers supported acceleration, as they saw that it provided students with more challenging content. However, they emphasised the need for individual variation in the implementation of acceleration and careful consideration of students’ social-emotional maturity. In Rogers’s (2015) research synthesis, single-subject acceleration produced moderate academic and psychological effects, while the effect on social adjustment was practically zero.

Southern and Jones (2015) specify five dimensions along which acceleration practices differ: pacing (i.e., rate of instruction), salience (i.e., the visibility of acceleration to others), peers, access, and timing. Some of these dimensions are highly relevant in the context of this study, namely lower secondary school science. Regarding pacing, students need sufficient mathematics skills to support their accelerated science instruction, and teachers must credibly assess learning before allowing accelerated students to proceed to the next level of the curriculum (Southern & Jones, 2015). Salience, in turn, is related to the elitism sometimes associated with acceleration practices. However, it may be possible to adjust the visibility to others; for example, making self-paced instruction widely available or labelling it modestly makes it less prominent than offering the option only to a few students or labelling it grandly (Southern & Jones, 2015). Regarding access, schools may need better resources to support accelerated students’ advanced laboratory work (Southern & Jones, 2015). Regarding timing, recommendations have been made in the literature to time grade skipping to natural curricular or administrative breaks, such as the transition between primary and lower secondary school; nevertheless, there is no systematic comparative research on students who have skipped grades at different times of the year or at different grade or school levels (Southern & Jones, 2015).

The literature identifies various reasons for the underutilisation of acceleration. Some educators equate acceleration primarily with grade skipping (Hoogeveen, 2015; A. Robinson et al., 2007) or early entrance (Hoogeveen, 2015). Limited understanding of asynchronous student development, personal bias, uneven class sizes, and transportation issues may make some educators and administrators reluctant to implement subject acceleration (Riegel & Behrens, 2022). These various barriers are context-dependent but important given that school is a context that plays a critical role in supporting talent development for gifted students (Gagné, 2004, 2021). Persistent concerns also relate to the misconception that acceleration has a negative impact on students’ social-emotional well-being (Assouline et al., 2021; Cross et al., 2015; A. Robinson et al., 2007), thus emphasising educators’ central catalytic role—and their knowledge of gifted education—in either supporting or hindering talent development (Gagné, 2004, 2021). Inclusive education has also been criticised for neglecting the needs of gifted students, making it important to examine how gifted students are served in inclusive settings (Tirri & Laine, 2017); this also applies to accelerative practices. Although evidence on acceleration in inclusive education is scarce, Dare and Nowicki (2023) point out that socially inclusive schools may in fact provide a more accepting environment for accelerated students, because such environments value diversity and individual differences by default.

Reasons for not using acceleration specifically for students gifted in science, technology, engineering, and mathematics (STEM) have also been identified (Assouline & Lupkowski-Shoplik, 2010; Ihrig & Degner, 2015). These include: (a) concerns about negative academic effects (e.g., students are not ready to study abstract concepts; there will be gaps in understanding; students will run out of courses before finishing school); (b) concerns about well-being (e.g., students will burn out); (c) differentiated instruction (e.g., teachers have been offered in-service training in differentiated instruction and therefore acceleration is not needed); (d) enrichment (e.g., enrichment and extracurricular activities are sufficient to serve gifted students); (e) assumptions of insufficient giftedness (e.g., a student makes mistakes in computational tasks or does not earn 100% on all tests); and (f) assumptions of sufficient acceleration (e.g., the belief that one accelerative measure is sufficient for every gifted student and there is no need for additional options). A substantial body of research has disproved these claims and misunderstandings (see Assouline & Lupkowski-Shoplik, 2010; Ihrig & Degner, 2015).

As a context-dependent notion, acceleration can be viewed as a form of differentiated instruction (Laine et al., 2025; Tomlinson, 2014). Differentiated instruction is the pedagogical foundation of all basic education in Finland, concerning progress, depth, extent, rhythm, and ways of learning (Finnish National Agency for Education, 2025). In Sweden, the latest national curriculum for compulsory education (Swedish National Agency for Education, 2024) does not explicitly use the term differentiated instruction but rather ‘adaptation’ of teaching; however, it emphasises the core principles of differentiated instruction, which include considering students’ individual needs and responding to them in appropriate ways (Huss, 2023; Swedish National Agency for Education, 2024).

### 1.2. The Context of the Study

Education systems in both Finland and Sweden emphasise equality, equity, and inclusion (Barow & Berhanu, 2021; European Agency for Special Needs and Inclusive Education, 2026; Frønes et al., 2020; Hammerness et al., 2017), although increasing liberalism and marketisation potentially threaten these aims. In Sweden, devolution to municipality level has led to decreased national consistency and equity (Barow & Berhanu, 2021; Magnússon, 2020; Paulsrud, 2022). Both countries pay special attention to the needs of low-achieving students and those with learning disabilities (European Agency for Special Needs and Inclusive Education, 2026; European Commission, 2025a; Laine & Tirri, 2021; National Agency for Special Needs Education and Schools, 2026). At the same time, gifted students have received significantly fewer services targeted to their special needs (Tirri & Kuusisto, 2013; Westling Allodi, 2014). In the Nordic countries, there is a general disapproval of highlighting exceptionality (Furnes & Jokstad, 2023; Tirri & Margrain, in press). Such an attitude is visible, for example, in the lack of public support for psychological testing for gifted identification; families must generally seek and pay for such services themselves, which places them in an inequitable position compared with students with learning disabilities. Nordic countries are signatories to the Salamanca Statement, an international commitment to inclusion—which explicitly mentions gifted children (United Nations Educational, Scientific and Cultural Organization, 1994). According to Gagné’s (2004, 2021) DMGT, cultural milieu is one of the central environmental catalysts in the development of giftedness. Therefore, the following section provides a more detailed description of the education systems and relevant provisions in both countries.

In Finland, compulsory education includes pre-primary education, basic education (i.e., grades 1–6, commonly called primary education, and grades 7–9, commonly called lower secondary education), and upper secondary education among children and youths between 6 and 18 years of age (Finnish National Agency for Education, 2026); this study focuses on lower secondary education (grades 7–9). Education is publicly funded and free of charge from pre-primary to higher education (Ministry of Education and Culture, n.d.). Basic education comprises grades 1–9, and children start grade 1 in the year they reach the age of seven (Finnish National Agency for Education, 2026). Children must also participate in pre-primary education in the year preceding the start of compulsory education (Ministry of Education and Culture, n.d.). Specialised schools are rare (Tirri & Kuusisto, 2013), and the few that exist receive public funding (European Commission, 2025b). There are no national high-stakes assessments in Finnish basic education (Kupiainen et al., 2009).

Neither Finnish school legislation nor the national core curricula include a definition of giftedness or criteria for identifying it, nor does the legislation explicitly mention gifted students or recognise them as a group with special needs. However, the legislation acknowledges individual differences and guides schools to provide education ‘according to the pupil’s age and capabilities and so as to promote healthy growth and development in the pupil’ (Basic Education Act 628, 1998, Chapter 1, Section 3, Amendment 477/2003). A child can start basic education one year earlier than stipulated; psychological and, where necessary, medical tests must be conducted to show the child’s capacity to manage schooling at an earlier age (Basic Education Act 628, 1998, Chapter 7, Section 27). The legislation also allows flexible arrangements, for example in situations where a student has prior knowledge and skills corresponding to the objectives set in the curriculum (Basic Education Act 628, 1998, Chapter 4, Section 18). A student can skip a grade or pursue objective-based studies (i.e., studies that do not follow a grade-based syllabus) according to a personal study plan (Finnish National Agency for Education, 2025). Permanent ability grouping is prohibited (Finnish National Board of Education, 1985). Gifted students were mentioned for the first time in 2014 in the national core curriculum for basic education (Finnish National Agency for Education, 2014). The document also included expressions such as ‘skilful pupils’ and ‘pupils who are advancing faster’, and it introduced some general-level suggestions on how to support such students. However, even today, identifying and serving the needs of gifted students depends heavily on individual teachers (Laine & Tirri, 2021).

In Sweden, basic education includes ten grades (0–9), with children starting the compulsory preschool class (grade 0) in the year they reach the age of six (European Commission, 2024). Compulsory education is regulated nationally through the Education Act and spans nine years of schooling, typically beginning the year a child turns seven, though flexibility allows starting between ages six and eight according to parental preference (Eurydice, 2024). A three-year upper secondary education is voluntary (Swedish National Agency for Education, n.d.-b). Municipalities are responsible for providing preschool classes, compulsory school, Sami school, and special schools, and compulsory education is embedded within a national framework emphasising equal access and age-appropriate learning (Eurydice, 2023, 2024). Alongside municipal schools, Sweden permits independently operated schools that receive public funding, a system documented in academic analyses of Swedish educational governance (Norlin, 2024); today, approximately one in five compulsory schools is independently operated (Swedish Institute, 2026). Most compulsory schools have a general orientation, but some have a specific orientation (e.g., sports, culture, or religion) or use a particular pedagogical approach (Swedish National Agency for Education, n.d.-a). While mandatory national assessment systems exist (Swedish Institute, 2026), Sweden does not operate high-stakes examinations comparable to some international systems, and assessment practices are guided by national learning goals rather than ranking-oriented testing.

Swedish legislation does not explicitly define giftedness or outline identification criteria for gifted students, a situation shaped historically by the egalitarian education ethos (Svedin, 2023). However, the national curriculum for compulsory education requires schools to adapt instruction to each student’s capabilities and needs (Swedish National Agency for Education, 2024). To support teachers in addressing gifted students’ needs, the Swedish National Agency for Education (2025) has provided a handbook with online resources. In 2010, legislation stipulated that students who easily achieve the minimum required level of knowledge and skills must be given guidance and stimulation to enable them to progress further in their learning (Education Act 800, 2010, Chapter 3, Section 2). A student can also skip a grade, based on the principal’s decision, provided that they have good prospects for participating in education at the higher grade level and that the student’s guardian consents (Ordinance Amending the Compulsory School Ordinance 1619, 2022, Chapter 4, Section 7). The Swedish National Agency for Education has also established advanced programmes in STEM subjects, the social sciences, and the humanities in several geographically dispersed upper and lower secondary schools (Westling Allodi & Szabo, in press), but in practice student selection is based not on high ability but on interest (Dodillet, 2019). Overall, although the 1994 curriculum reforms opened up the possibility of targeted support for high-ability learners, subsequent curricular frameworks have become less explicit, placing responsibility for identifying and supporting gifted students largely in the hands of individual teachers and schools (Svedin, 2023; Norlin, 2024). Research indicates increasing recognition of the need for differentiated instruction and flexible pedagogical arrangements to meet the needs of high-ability learners in mixed-ability classrooms, though implementation varies across municipalities (Ardenlid, 2025).

### 1.3. Epistemological and Methodological Context

This study’s epistemological foundation is rooted in pragmatism, a problem-centred and pluralistic research paradigm oriented toward real-world practices (Creswell & Plano Clark, 2011) and human experience (Morgan, 2014). Pragmatism is often associated with mixed methods research (Creswell & Plano Clark, 2011; Morgan, 2014). Methodological pragmatism is a flexible paradigm for conducting educational research in which the selection of methods is determined pragmatically—that is, based on the needs of the research in question (Foster, 2024). Accordingly, research grounded in such a paradigm may employ combinations of qualitative or quantitative methods, including mixed methods approaches (Johnson et al., 2007). This study employed mixed methods to gain both broad and in-depth insight into the research topic. More specifically, we adopted a parallel mixed design (Tashakkori & Teddlie, 2009), in which the quantitative and qualitative data were collected simultaneously, analysed separately, and subsequently synthesised into an integrated outcome.

The study is characterised as exploratory research complemented with inferential group comparisons. An exploratory research approach allows researchers to develop insights in areas where existing knowledge is limited or not yet sufficiently understood (Stebbins, 2001). Its purpose is not to test predetermined hypotheses but to generate initial understanding, identify key concepts and relationships, formulate questions for further investigation, and produce inductively derived generalisations about the group, activity, or situation under study (Stebbins, 2001).

This approach was considered beneficial for the purposes of the present study, as there is very limited prior knowledge regarding teachers’ acceleration practices in the Nordic inclusive context. In addition to producing descriptive quantitative and qualitative findings, we complemented our analyses with quantitative group comparisons. In particular, it was considered important to examine teachers’ practices separately across the two countries and between teachers with or without knowledge of gifted education. Such knowledge is valuable for potential future instrument development that may require context-specific adjustments.

## 2. Materials and Methods

### 2.1. Participants

Physics and chemistry teachers at the lower secondary level (i.e., grades 7–9) were contacted through multiple channels and invited to complete an online survey anonymously. A pragmatic approach to recruitment was used, relevant to each country’s context. In Sweden, education administrators from all municipalities and most independent schools were contacted, whereas in Finland, the largest municipalities within each Regional State Administrative Agency in mainland Finland, along with most privately run schools, were approached. Participation was voluntary at both the administrative and teacher levels: education administrators and principals decided whether to distribute the survey to schools and teachers, and teachers independently chose whether to participate. Additionally, teachers were recruited through science teacher associations, science education centres, science teacher groups on social media, and other networks. Consequently, the samples in both countries constitute convenience samples.

The sample (*N* = 160) comprised 96 (60%) teachers from Finland and 64 (40%) teachers from Sweden. Eighty-eight (55%) respondents identified themselves as female and 71 (44%) as male; the proportion of female teachers was 53% in Finland and 58% in Sweden. The vast majority (*n* = 153, 96%) reported being qualified subject teachers. Physics was a major subject for 67 (42%) teachers, while 65 (41%) had a major in chemistry. In addition, 75 (47%) reported a major in mathematics, 29 (18%) in biology, 25 (16%) in technology, 4 (3%) in computer science, and 20 (13%) in other subjects, such as astronomy, philosophy, or biochemistry. Teachers could report more than one major; some variation in the content of subject-teacher education has occurred across countries and over time. A similar range of subjects, with a few teachers also mentioning geography, were reported as minor subject studies.

The sample included teachers with a wide age range: less than 30 years (*n* = 17, 11%), from 30 to 40 years (*n* = 42, 26%), from 41 to 50 years (*n* = 40, 25%), from 51 to 60 years (*n* = 39, 24%), and more than 60 years (*n* = 22, 14%). There were respondents from 14 counties (out of 19 in total) in Finland and from 16 counties (out of 21 in total) in Sweden. The sample also included teachers with a wide range of experience teaching at the lower secondary level: less than one year (*n* = 6, 4%; *n*_FI_ = 5, 5%; *n*_SE_ = 1, 2%); from 1 to 5 years (*n* = 36, 23%; *n*_FI_ = 21, 22%; *n*_SE_ = 15, 23%); from 6 to 10 years (*n* = 28, 18%; *n*_FI_ = 20, 21%; *n*_SE_ = 8, 13%); from 11 to 20 years (*n* = 44, 28%; *n*_FI_ = 29, 30%; *n*_SE_ = 15, 23%); from 21 to 30 years (*n* = 38, 24%; *n*_FI_ = 15, 16%; *n*_SE_ = 23, 36%); and more than 30 years (*n* = 8, 5%; *n*_FI_ = 6, 6%; *n*_SE_ = 2, 3%).

Fifteen teachers were not working at the lower secondary level at the time of responding, but they had prior experience teaching at that level. These respondents were included in the analysis because the survey instructed participants to answer only in the context of lower secondary education. This instruction was also repeated in the invitation letter and in the consent form; therefore, it was clear to the respondents that they were expected to answer the questions only with regard to grades 7–9. There were also respondents who taught at both the lower and upper secondary levels (*n* = 20), at both the elementary and lower secondary levels (*n* = 1), or at the elementary, lower secondary, and upper secondary levels (*n* = 1).

Approximately half of the participants (*n* = 79, 49%) in the entire sample reported having some knowledge of gifted education (*n*_FI_ = 46, 48% of the Finnish teachers; *n*_SE_ = 33, 52% of the Swedish teachers). More specifically, 35 (22%) respondents (*n*_FI_ = 22, *n*_SE_ = 13) had read information about gifted education in national education administration documents. Eighteen (11%) teachers (*n*_FI_ = 7, *n*_SE_ = 11) had participated in GE-related in-service training provided by their employer. For 16 (10%) teachers (*n*_FI_ = 10, *n*_SE_ = 6), such training was provided by a unit/institution other than their employer. Forty-two (26%) teachers (*n*_FI_ = 27, *n*_SE_ = 15) reported having studied gifted education independently (e.g., by reading articles or books), and 34 (21%) teachers (*n*_FI_ = 17, *n*_SE_ = 17) had learnt about gifted education in other ways. Teachers with knowledge of gifted education also evaluated the time they had spent on learning about the topic, with the medians being 10 h (*Md*_FI_ = 9 h, *Md*_SE_ = 10 h) for in-service training, 25 h (*Md*_FI_ = 22 h, *Md*_SE_ = 30 h) for independent study, and 5 h (*Md*_FI_ = 5 h, *Md*_SE_ = 7.5 h) for other ways.

### 2.2. The Instrument

The survey questionnaire (see Appendix A) was created in Survey&Report and comprised closed- and open-ended items as well as background questions. The questionnaire formed part of a wider survey that also included sections on differentiated instruction and grouping; these will be addressed in a separate article.

For the present article, the questions were designed on the basis of the literature on accelerative practices. Given the large variety of such practices (e.g., Southern & Jones, 2015), we focused on those considered most relevant in the Nordic basic education context: early entrance, grade skipping, subject-based grade-level acceleration, and curriculum compacting (Laine et al., 2025).

We used multiple-choice questions to examine whether teachers had taught students who were accelerated via early entrance, grade skipping, or subject-based acceleration, and to determine how many such students they had taught. Open-ended questions were posed to examine how these forms of acceleration had affected teachers’ instructional practices.

We included five multiple-choice items associated with curriculum compacting. However, the term curriculum compacting was not used explicitly, as we assumed it would be unfamiliar to most teachers (even if they might use the practice). Instead, we asked about diagnostic assessment, higher-grade-level content, project work during school hours and in free time, and other arrangements. Based on the first authors’ prior professional experience in lower secondary education, we expected that adding content from a higher grade level would be implemented more frequently than the other options; we therefore added four clarifying items to gain a deeper understanding of this practice (these were shown to respondents if they gave a positive response to the main item addressing higher-grade-level content). For all these individual items, seven response alternatives were provided on a Likert-type frequency scale (1 never, 2 very rarely, 3 about once a semester, 4 about once a month, 5 about once a week, 6 a few times a week, 7 every day).

To obtain a broader perspective on practices related to curriculum compacting, we also used the Curriculum Modifications (CM) scale of the Classroom Practices Survey–Revised (CPS-R) by Pereira et al. (2021); permission to use the instrument was obtained. The original survey was developed by Archambault et al. (1993) to assess how teachers accommodate instruction for gifted students in regular classrooms. Pereira et al. (2021) updated the survey and validated the revised version in the US context. The CM scale comprises 11 items that we considered relevant to aspects of curriculum compacting—assessment strategies, eliminating or adding content, and using tiered lesson plans (see Appendix A). The scale includes six response options rated on a Likert-type frequency scale (0 never, 1 once a month or less frequently, 2 a few times a month, 3 a few times a week, 4 daily, 5 more than once a day).

Besides these closed-ended items, we provided space for teachers to describe in their own words any other accelerative arrangements (i.e., teacher-defined forms of acceleration) they implemented in their teaching.

To gain insight into the time- and material-related resources for acceleration, we adopted two items from Schroth’s (2007) study; permission was obtained. Five response options were provided on a Likert-type scale (1 strongly disagree, 2 disagree, 3 I don’t know, 4 agree, 5 strongly agree).

Finally, to obtain an overall picture of the frequency of acceleration, we asked respondents to indicate how often they used acceleration for a gifted student. One multiple-choice question with a seven-point Likert-type scale was included (1 never, 2 very rarely, 3 in at least 25% of the lessons, 4 in at least 50% of the lessons, 5 in at least 75% of the lessons, 6 in almost every lesson, 7 in every lesson).

The background information section included questions about age, self-identified gender, place of residence (county and city/municipality), the language the respondent mostly speaks at home, education, teacher qualification(s), teaching experience in years (in total and at the lower secondary education), major and minor subject(s), the school level at which the respondent teaches, whether the respondent has some knowledge of gifted education, and details on how such knowledge was obtained (e.g., participation in in-service training, independent reading). Due to the lack of definition of giftedness, the following guideline was provided at the beginning of the survey: ‘In Finland/Sweden, there is no official national policy definition of giftedness. Use your experience and knowledge as a teacher to think about students with potential or exceptional abilities or skills when answering the following questions.’

The first version of the survey was written in English and reviewed by two experts: a researcher with extensive experience in Nordic gifted education research and an experienced teacher-researcher in science education. A few terminology-related adjustments were made based on these reviews. The final version was translated into Finnish and Swedish using a back translation procedure by professional language services. Next, two native Finnish-speaking science teachers—one of whom is also a teacher educator and researcher in science education—pilot-tested the Finnish translation. The Swedish translation was also pilot-tested by two experts: a researcher in gifted education with a science teacher background, and another researcher in gifted education, both native Swedish speakers. Some changes in wording were made to both translations based on their comments. An additional Swedish version was also produced for the Swedish-speaking minority in Finland; it differed from the original Swedish version in a few background information details. Finally, the authors—one a native Finnish speaker fluent in English and Swedish, the other a native English speaker fluent in Swedish—compared the English, Finnish, and two Swedish versions to ensure that the translations corresponded with each other.

### 2.3. Data Analysis

Cross-tabulation with chi-squared analysis was conducted to examine whether categorical data differed between the two countries. For the individual items rated on a Likert-type scale, descriptive statistics were produced and non-parametric Mann–Whitney *U*-tests were used to examine whether differences were significant between countries, genders, and teachers with or without knowledge of gifted education. A Kruskal–Wallis one-way analysis of variance was used to examine differences between respondents with varying lengths of teaching experience.

Principal component analysis (PCA) was used to test the structure of the CM-scale items—in other words, to confirm whether the items constituted a single scale. However, more than one principal component emerged, and certain items were removed iteratively. Cronbach’s alpha was used to test the reliability of the final components; key figures on PCA are presented in Section 3.4.2. Spearman’s rho correlation analysis was applied to examine how the components were related to each other and to the background variables. We analysed the statistical significance of the observed differences by means of independent-samples *t*-tests; parametric tests are shown to be robust with Likert-type data when sum scores are used (Carifio & Perla, 2008; Norman, 2010). SPSS version 30.0 was used for the statistical analyses.

Regarding the open-ended data, we used an inductive content analysis approach to classify the responses into categories (Elo & Kyngäs, 2008) and reported the frequency of each category. In inductive content analysis, categories are derived from the data; the aim is to produce both a concise and a broad description of the topic, and the result is an emergent category or concept structure (Elo & Kyngäs, 2008).

The process began with a preparation phase in which the first author divided the responses into units of analysis within each open-ended question. A unit of analysis constituted a meaning unit, with lengths varying from one word to a few sentences. For example, regarding the question about teacher-defined forms of acceleration (i.e., other arrangements teachers described in their own words), the response ‘differentiating the difficulty level and additional instructional sessions/special small groups’ was divided into three units of analysis: ‘differentiating the difficulty level’, ‘additional instructional sessions’, and ‘special small groups.’ After repeatedly examining the data, the first author assigned a code to each unit of analysis. For example, the three abovementioned units of analysis were labelled with the codes ‘Adjusting difficulty level’, ‘Additional teaching’, and ‘Small groups’. Adhering to the systematic procedures of qualitative content analysis, all data were coded (Schreier, 2012); the authors did not selectively include or exclude data based on perceived relevance. The first author then grouped together related codes to form categories. For example, codes such as ‘Adjusting difficulty level’, ‘Problem-solving challenges’, and ‘Widening and deepening’ were grouped under the category ‘More challenging tasks’. Similarly, codes such as ‘Small groups’ and ‘A group based on interest and giftedness’ were grouped under the category ‘Grouping’. Some categories were further combined; for example, the categories ‘Timetable adjustment’ and ‘Project work’ were grouped under the category ‘Miscellaneous practices’. Finally, the first author established the final category structure and created a code book in which each category was accompanied by one to ten example codes.

The second author independently coded the Swedish-language data—a total of 107 units of analysis—using the codebook developed by the first author, by assigning each unit to one of the final categories. Inter-rater reliability was assessed using Cohen’s kappa, calculated separately for two main sections of the open-ended data. First, regarding the effects of early entrance, grade skipping, and subject-based acceleration on teachers’ instructional practices, the kappa value (κ = 0.89) indicated agreement beyond the substantial level (Landis & Koch, 1977); all these subsections had the same category structure comprising five categories. Second, a similar level of agreement was reached regarding teacher-defined forms of acceleration (κ = 0.94); this section had a separate category structure comprising seven categories. The authors discussed any disagreements until a common interpretation was reached. Next, the first author independently reviewed the initial coding of the Finnish-language data and calculated the frequency of each category for the entire dataset. The first author also translated the selected quotations representative of each category’s content from Finnish and Swedish into English. To preserve the original meaning, the translations were kept as literal as possible. We analysed the qualitative data in Excel and calculated Cohen’s kappa values in SPSS version 30.0.

### 2.4. Ethics

The study forms part of a larger research project that underwent ethical review in accordance with the guidelines of Karlstad University. The approval is recorded under reference number HS 2025/992. Based on a decision by the research ethics advisor at Karlstad University, the study did not require national ethical review by the Swedish Ethical Review Authority under the Swedish Ethical Review Act.

## 3. Results

Table 1 presents the items and corresponding results regarding teachers’ experience of teaching accelerated students, including early entrance, grade skipping, and subject-based acceleration. Table 2 complements these results by indicating how many such students the teachers reported having taught. The results from further analyses are presented separately for early entrance, grade skipping, and subject-based acceleration in Section 3.1, Section 3.2 and Section 3.3. The results regarding curriculum compacting are presented in Section 3.4. Section 3.5 addresses teacher-defined forms of acceleration, and Section 3.6 presents teachers’ views on the resources available for acceleration. Finally, Section 3.7 addresses the overall frequency of acceleration.

### 3.1. Early Entrance

Finnish and Swedish teachers differed in their experience of having early-entrance students (Table 1). In the Finnish sample, 51% of respondents reported that they did not know whether their students had entered school earlier, whereas in the Swedish sample only 13% were uninformed. In turn, one quarter of the Finnish sample stated that they had taught early-entrance students, while in Sweden the proportion was 57%. The result of the chi-squared test of independence showed a statistically significant association between country and experience, χ^2^ (2, *N* = 158) = 25.80, *p* < 0.001. The effect size, as measured by Cramér’s *V* (*V* = 0.40), indicated a medium-to-large effect (Cohen, 1988). Post hoc comparisons showed that the countries differed significantly in the proportions of respondents selecting the Agree response category (i.e., respondents reported having taught early-entrance students) and the No such information about students response category (i.e., respondents reported not having information about students’ early entrance), whereas no significant between-country difference was found for respondents selecting the Disagree response category (i.e., respondents reported not having taught early-entrance students).

Teachers were asked how many early-entrance students they had taught (Table 2). The numbers in Finland are small: only eight (8%) teachers reported five or more such students, and 14 (15%) reported from one to three such students. In Sweden, almost one-third (*n* = 20, 31%) reported five or more early-entrance students, and 15 (23%) had experience of teaching one to four such students.

It could be expected that the number of these students would increase with the length of teaching experience. However, cross-tabulation (in the entire sample) showed that some of the less-experienced teachers reported several early-entrance students, while many highly experienced teachers were aware of having taught only a few such students. To meet the requirements for applying chi-squared statistics, we regrouped the teaching-experience categories as 10 years or less, 11–20 years, and 21 years or more. We also regrouped the categories for the number of early-entrance students taught as three students or fewer and four students or more (Table A1 in Appendix B). No significant association between the variables was found, χ^2^ (2, *N* = 57) = 5.68, *p* = 0.058, suggesting that teachers do not encounter early-entrance students on a systematic basis.

An open-ended question was posed to teachers about the effects of early entrance on their teaching practices. Of the 54 units of analysis identified from the responses, 22 were from Finland and 32 from Sweden. Five categories emerged in the inductive content analysis. The largest category, No effects, with 36 (67%) units of analysis, included statements about a total lack of observed effects (e.g., ‘I haven’t noticed a difference’ and ‘no, got to know afterwards’) and statements referring to hardly any influence (e.g., ‘Usually not in any way, because the acceleration has already taken place’ and ‘Not particularly much, regardless of the student’). The Challenges category included eight (15%) problematic experiences, all related to student characteristics, for example ‘socially very different‘, ‘One comes to mind right now, and regarding this student, I think the decision has been completely wrong. In ninth grade, the level of [the student’s] competence is practically non-existent’, and ‘Sometimes adaptation is needed based on the level of maturity, which can make things especially difficult.’ The Special arrangements category included six (11%) descriptions of adjustments teachers had made for their early-entrance students, including ‘I have offered the student more challenging tasks’ and ‘I always try to make sure that there are different levels of difficulty in the instruction.’ There was only one short mention, ‘good’, in the Positive effect category, and three miscellaneous statements in the Other views category, one example being ‘Many such students are not ahead of the others when they reach the lower secondary level’.

### 3.2. Grade Skipping

A similar pattern was observed for grade skipping as for early entrance. Half of the Finnish teachers stated that they did not have information on whether their students had skipped a grade, and only 13% reported having taught such students; in Sweden, the situation was the opposite (Table 1). A chi-squared test of independence revealed a significant association between country and experience, χ^2^ (2, *N* = 156) = 31.12, *p* < 0.001, with Cramér’s *V* (*V* = 0.45) indicating a moderately strong association. Post hoc comparisons showed that the countries differed significantly in the proportions of respondents selecting the Agree response category (i.e., respondents reported having taught grade-skipping students) and the No such information about students response category (i.e., respondents reported not having information about students’ grade skipping), whereas no significant between-country difference was found for respondents selecting the Disagree response category (i.e., respondents reported not having taught grade-skipping students).

Overall, the reported number of grade-skipping students was small (Table 2). Cross-tabulation showed that less experienced teachers reported one to three such students, but a wider range was observed among more experienced teachers (Table A2 in Appendix B). However, the criteria for applying a χ^2^ test were not met, even after combining categories, and we could not confirm whether the number of grade-skipping students increased with teaching experience.

Teachers were asked to describe how students’ grade skipping had affected their teaching practices. Thirty-three units of analysis—12 from Finland and 21 from Sweden—were identified from the open-ended responses. Approximately half of the units of analysis in both countries showed no perceived effects, and none of the respondents reported any challenges. Eleven (33%) units of analysis—nine in Sweden and two in Finland—addressed special arrangements, for example, ‘Experimental skills and metacognitive thinking needed guidance and development of skills’, ‘less repetition’, and ‘In-depth assignments based on the student’s interests.’ There were also two mentions of positive effects, one from each country, such as ‘In grade 7, the student understood chemistry at a grade 12 level using memory and logical reasoning.’ The Other views category included two units of analysis from Finland, one of which was ‘Teaching practices regarding gifted students, in particular, are based on [the teacher’s] knowledge of their group and their students and not on age or preconceptions.’

### 3.3. Subject-Based Acceleration

The majority (ca. 60%) of teachers in both countries stated that they had not taught subject-accelerated students (Table 1). More teachers in Finland (23%) than in Sweden (5%) were unaware of whether subject-based acceleration had been implemented for their students. Only 14% of Finnish teachers reported having taught such students, whereas in Sweden the proportion was 37%. A chi-squared test of independence showed that the association between country and experience was significant, χ^2^ (2, *N* = 158) = 17.14, *p* < 0.001, with Cramér’s *V* (*V* = 0.33) indicating a medium association; post hoc comparisons indicated significant differences between the countries in the Agree and No such information about students categories, but not in the Disagree category.

In general, the reported number of subject-accelerated students was small (Table 2). Cross-tabulation of teaching experience and the number of students showed that, in all three experience groups, there were teachers with a small number and teachers with a larger number of students (Table A3 in Appendix B). The criteria for applying a χ^2^ test were not met, even after combining categories, and we could not confirm whether the number of students in subject-based acceleration increased with teaching experience.

Thirty-five units of analysis—12 from Finland and 23 from Sweden—were derived from teachers’ open-ended responses regarding the effects of subject-based acceleration. In both countries, approximately two-thirds addressed special arrangements, for example, ‘We looked for a 9th grade physics group in the timetable for a student so that the student could do experimental work’, ‘[the student] could take courses from an upper secondary school with support from that school’, and ‘The student has been allowed to work with their own material alongside the rest of the class.’ Five (Swedish) teachers (14%) reported positive effects, such as ‘One is more engaged in teaching’ and ‘Only positive!’ Teachers in both countries mentioned only a few challenges, such as ‘Within the framework of a lesson, it is not very fruitful to teach physics at two grade levels, or physics and chemistry at the same time (unless it involves independent studies).’ In addition, two units of analysis showed no effects and two addressed other views.

### 3.4. Curriculum Compacting

#### 3.4.1. Individual Items

The frequencies of the individual items on curriculum compacting for the entire sample are presented in Table 3. For the four main items, more than 60% of respondents reported that they had never or very rarely implemented the practice in question. As expected, the most frequently used practice was instructing a gifted student to study content from a higher grade level (*M* = 2.50, *SD* = 1.44). Results from the four clarifying items indicated that students most typically study higher-grade-level content as part of their ordinary instruction in their regular group (*M* = 3.04, *SD* = 1.48). However, the use of this practice—as with all the other practices in Table 3—is infrequent; few teachers reported implementing them on a weekly or even monthly basis.

Mann–Whitney *U*-tests were applied to examine whether the results differed between the two countries, between teachers with and without knowledge of gifted education, and between teachers’ genders. The countries differed regarding diagnostic assessment, studying content from a higher grade level, and working independently on a challenging project during school hours, with Swedish teachers reporting the use of such practices more frequently than their Finnish counterparts (Table A4 in Appendix B). Teachers with knowledge of gifted education reported implementing most of the practices more frequently than teachers without such knowledge did (Table A5 in Appendix B), irrespective of whether they were from Finland or Sweden. No statistically significant differences were found between male and female teachers on any of the items.

We applied a Kruskal–Wallis one-way analysis of variance to examine whether the results differed between respondents with different lengths of teaching experience at the lower secondary level. The three experience categories used in the analysis were 10 years or less, 11–20 years, and 21 years or more. A statistically significant difference (*H*(2) = 6.895, *p* = 0.032, η_H_^2^ = 0.05) between the experience groups was found only for the last clarifying item. Pairwise comparisons showed that the least experienced teachers (*Md* = 2, *IQR* = 2, *MR* = 67.71, *n* = 41) reported instructing gifted students to study higher-grade-level content independently in an out-of-school setting more frequently than the most experienced teachers (*Md* = 2, *IQR* = 2, *MR* = 50.61, *n* = 38), *Z* = 2.451, *p* = 0.043, with the effect size (*r* = 0.28) indicating a small-to-medium effect (Fritz et al., 2012). However, the difference is practically negligible, as this practice was applied very infrequently in both groups (less than once a semester).

#### 3.4.2. Curriculum Modification (CM) Scale

The results of the Shapiro–Wilk tests of normality revealed that none of the items in the CM scale were normally distributed. We therefore opted for PCA (rather than factor analysis) to determine which items were connected in our sample. Direct Oblimin rotation was used on the assumption that the principal components would correlate. The first analysis produced three principal components. We removed three items with the lowest extraction communalities iteratively, but the resulting structure with eight items still produced three principal components; the first comprised three items related to assessment, and the second comprised three items related to curriculum adjustments. However, the two remaining items did not form a coherent entity and also loaded on two components; these items were removed from further analyses.

The final solution consisted of two principal components with eigenvalues exceeding Kaiser’s rule of 1 and explaining 69.41% of the variance (Table 4). The Kaiser–Meyer–Olkin measure (*KMO* = 0.707) indicated a middling level of sampling adequacy (De Vaus, 2002), and Bartlett’s test of sphericity was significant (*p* < 0.001). The extraction communalities (ranging from 0.56 to 0.80; see Table 4) were moderately high, and the component loadings (ranging from 0.66 to 0.89; see Table 4) were above the acceptable threshold of 0.32 (Tabachnick & Fidell, 2014). Two sum variables were created based on the two principal components, with three items in each (Table 4). Cronbach’s alpha was used to assess the internal consistency of these new scales—titled Assessment and Content adjustment. Both scales had alpha coefficients (α = 0.827, α = 0.702, resp.) exceeding the commonly used criterion of 0.70 (De Vaus, 2002).

Correlation analysis was conducted to evaluate how the scales were related to each other and to background variables. According to the Shapiro–Wilk test of normality, only the Content adjustment scale was normally distributed, and a nonparametric procedure with Spearman’s rho was adopted. As shown in Table 5, the scales correlated with each other (rho = 0.25, *p* < 0.01). Country correlated with the Assessment scale (rho = −0.37, *p* < 0.01), indicating that Finnish teachers reported using assessment-related practices more frequently than their Swedish counterparts did. In addition, knowledge of gifted education correlated with the Assessment scale (rho = −0.23, *p* < 0.01) and the Content adjustment scale (rho = −0.27, *p* < 0.01), indicating that teachers with knowledge of gifted education reported using such practices more frequently than teachers without knowledge of gifted education did, across both Finland and Sweden. Independent-samples *t*-tests showed that all these differences were statistically significant: *M*_FI_ = 3.49 (0.99), *n* = 91, *M*_SE_ = 2.65 (1.06), *n* = 51, *t*(140) = 4.750, 95% CI = [0.491, 1.191], *p* < 0.001; *M*_with_ = 3.37 (1.11), *n* = 69, *M*_without_ = 2.88 (0.98), *n* = 66, *t*(133) = 2.706, 95% CI = [0.131, 0.845], *p* = 0.008; *M*_with_ = 2.43 (1.05), *n* = 76, *M*_without_ = 1.88 (0.92), *n* = 72, *t*(146) = 3.436, 95% CI = [0.238, 0.881], *p* < 0.001, with the effect sizes, as measured by Cohen’s *d*, indicating a large (*d* = 0.83), medium (*d* = 0.47), and medium (*d* = 0.56) effect, respectively (Cohen, 1988).

### 3.5. Teacher-Defined Forms of Acceleration

A multiple-choice item was included to examine how often teachers implemented accelerative practices other than those already mentioned in the survey, in other words, teacher-defined forms of acceleration. Of those responding (*N* = 152), the majority (*n* = 96, 63%) stated that they had never implemented such arrangements. Twenty-eight (18%) teachers reported implementing such practices very rarely, 12 (8%) about once a semester, and 12 (8%) about once a month. The remaining four teachers (3%) reported using them about once a week, a few times a week, or every day.

Based on Mann–Whitney *U*-tests, Swedish respondents (*Md* = 1, *IQR* = 2, *MR* = 86.55, *n* = 60) stated that they implemented such practices more frequently than their Finnish counterparts (*Md* = 1, *IQR* = 1, *MR* = 69.95, *n* = 92) did; *U* = 3363.00, *Z* = 2.641, *p* = 0.008, with the effect size (*r* = 0.21) indicating a small-to-medium effect (Fritz et al., 2012). Likewise, teachers with knowledge of gifted education (*Md* = 1, *IQR* = 2, *MR* = 80.27, *n* = 75) reported implementing such practices more frequently than teachers without knowledge of gifted education (*Md* = 1, *IQR* = 1, *MR* = 65.21, *n* = 70) did; *U* = 2080.00, *Z* = −2.498, *p* = 0.013, *r* = 0.21 (i.e., a small-to-medium effect). No statistically significant difference was found between male (*Md* = 1, *IQR* = 1, *MR* = 70.78, *n* = 66) and female (*Md* = 1, *IQR* = 1, *MR* = 80.05, *n* = 85) participants: *U* = 2460.50, *Z* = −1.506, *p* = 0.132, *r* = 0.12. We used a Kruskal–Wallis one-way analysis of variance to examine whether the results differed between respondents with different lengths of teaching experience. The *MR* (85.12) of the most experienced group (≥21 years), *n* = 65, was higher than that of the middle group (11–20 years), *MR* = 76.15, *n* = 41, and the least experienced group (≤10 years), *MR* = 70.62, *n* = 46, but the differences were not statistically significant: *H*(2) = 3.947, *p* = 0.139, η_H_^2^ = 0.01; *Md*_0–10_ = *Md*_11–20_ = *Md*_21+_ = 1; *IQR*_0–10_ = *IQR*_11–20_ = *IQR*_21+_ = 1.

Teachers were also asked to describe these forms of acceleration. Thirty-nine units of analysis—16 from Finland and 23 from Sweden—were identified in open-ended responses, with seven categories emerging in the analysis: (a) The More challenging tasks category included eight (21%) descriptions, such as ‘In addition to the instructional content intended for all students, some content from subsequent grade levels in the form of various tasks’ and ‘Differentiation of the difficulty level’. (b) The Additional instruction category included seven (18%) units of analysis—six from Sweden and one from Finland—such as ‘Coaching in leisure time’ and ‘Additional teaching’. (c) The Mathematics-related category included five (13%) comments about using acceleration in mathematics, such as ‘This concerns only mathematics, but … The student first got support from a university mathematics student in regular math classes…’. (d) The Independent studying category included four (10%) short statements, such as ‘Guided independent studies.’ (e) The Grouping category included two (5%) mentions, both from Sweden, such as ‘A group for particularly interested or gifted students.’ (f) The Miscellaneous practices category included nine (23%) mutually distinct statements, such as ‘[I] guided [the student], e.g., to study at another educational institution’, ‘Films related to the topic’, ‘Projects, e.g., “Framtidens forskare” [Future researchers]’, and ‘integration between subjects’. (g) The Critique category comprised four (10%) statements, all from Finland, that were critical of acceleration, for example, ‘in physics-chemistry, I have never encountered a student so advanced that they could develop a broad understanding of an in-depth theory so effortlessly that they would need additional topics to study’ and ‘There don’t seem to be all that many students in Finland these days whose instruction should be accelerated. I have only met one exceptionally gifted student in ten years. I don’t know whether we simply fail to identify them and then initiate differentiated instruction already in primary school, or where the problem lies. In my view, lower secondary school physics is already challenging in itself, and the topics are hardly studied in the lower grades at all, so even if a student is otherwise gifted, they don’t have any more competence in physics than anyone else.’

### 3.6. Resources for Acceleration

Teachers were asked to evaluate whether they had adequate planning time to accelerate instruction; of those responding (*N* = 155), the majority (*n* = 119, 77%) disagreed or strongly disagreed, while only 24 (15%) agreed or strongly agreed, and 12 (8%) stated that they did not know. On a scale of one to five, with higher values indicating agreement, the mean was *M* = 2.04 (*SD* = 1.13), 95% CI [1.86, 2.22]; the option ‘I don’t know’ was placed in the middle of the scale and given the value of 3.

We also asked whether teachers had access to the instructional materials necessary to accelerate instruction. Among the respondents (*N* = 155), the majority (*n* = 105, 68%) disagreed or strongly disagreed, 41 (26%) agreed or strongly agreed, and nine (6%) teachers stated that they did not know. Using the above-mentioned scale, the mean was *M* = 2.35 (*SD* = 1.29), 95% CI [2.15, 2.56].

To examine whether the countries differed on these two items, Mann–Whitney *U*-tests were used. No statistically significant differences were found in time resources (*U* = 2654.00, *Z* = −0.889, *p* = 0.374, *r* = 0.07) between Finnish (*Md* = 2, *IQR* = 2, *MR* = 80.46, *n* = 93) and Swedish (*Md* = 2, *IQR* = 1, *MR* = 74.31, *n* = 62) teachers, and the same applied to material resources: *U* = 2966.00, *Z* = 0.317, *p* = 0.751, *r* = 0.03; *Md*_FI_ = 2, I*QR*_FI_ = 3, *MR*_FI_ = 77.11, *n*_FI_ = 93; *Md*_SE_ = 2, *IQR*_SE_ = 2, *MR*_SE_ = 79.34, *n*_SE_ = 62.

### 3.7. Overall Frequency of Acceleration

Teachers assessed how often they used acceleration for a gifted student. Of those responding (*N* = 157), 42 (27%) stated that they never accelerate, and 81 (52%) stated they accelerate very rarely. Fourteen (9%) teachers reported using acceleration in at least one quarter of their lessons, eight (5%) teachers in at least half of their lessons, and eight (5%) teachers in at least three quarters of their lessons. In addition, four (3%) teachers reported using acceleration in almost every lesson. On a seven-point Likert-type scale, with higher values indicating frequent use, the mean was *M* = 2.18 (*SD* = 1.19), 95% CI [1.99, 2.37].

Mann–Whitney *U*-tests were applied to examine whether the results differed between the two countries, between teachers with and without knowledge of gifted education, and between genders. Swedish teachers (*Md* = 2, *IQR* = 1, *MR* = 89.95, *n* = 62) reported a higher implementation rate than their Finnish counterparts (*Md* = 2, *IQR* = 1, *MR* = 71.92, *n* = 95) did: *U* = 3617.5, *Z* = 2.631, *p* = 0.009, *r* = 0.21 (i.e., a small-to-medium effect). In the entire sample, teachers with knowledge of gifted education (*Md* = 2, *IQR* = 1, *MR* = 87.62, *n* = 77) reported more frequent implementation than teachers without such knowledge (*Md* = 2, *IQR* = 1, *MR* = 62.72, *n* = 73) did: *U* = 1877.5, *Z* = −3.823, *p* < 0.001, *r* = 0.31 (i.e., a medium effect). No statistically significant difference was found between male (*Md* = 2, *IQR* = 0, *MR* = 77.09, *n* = 68) and female (*Md* = 2, *IQR* = 2, *MR* = 79.59, *n* = 88) participants: *U* = 2896.0, *Z* = −0.373, *p* = 0.709, *r* = 0.03. We used a Kruskal–Wallis one-way analysis of variance to examine whether the results differed between respondents with different lengths of teaching experience; no statistically significant difference was found (*H*(2) = 1.696, *p* = 0.428, η_H_^2^ = 0.002) between the three experience groups: 10 years or less (*Md* = 2, *IQR* = 1, *MR* = 74.08, *n* = 68), 11–20 years (*Md* = 2, *IQR* = 0, *MR* = 81.98, *n* = 43), and 21 years or more (*Md* = 2, *IQR* = 0, *MR* = 83.49, *n* = 46).

## 4. Discussion

Psychology and gifted education research have provided strong evidence of the benefits of acceleration for gifted students (e.g., Assouline et al., 2015; Rogers, 2015; Wardman, 2017). However, our findings indicate that educators do not implement acceleration to a sufficient extent in Finland and Sweden—two Nordic countries with a strong emphasis on educational equality and inclusion. The results provide much-needed evidence on the implementation of acceleration from teachers’ perspectives and reveal a discrepancy between instructional practices and the opportunities offered by the education systems.

The reported overall frequency of acceleration was low in both countries, although somewhat higher in Sweden. More than one quarter of teachers stated that they never use acceleration, and more than half reported using it very rarely. These findings are not entirely surprising, given that giftedness is neither widely valued nor supported in either country (Mattsson & Bengmark, 2011; Persson, 2010; Sims, 2023; Tirri, 2022; Tirri & Margrain, in press). Nevertheless, the findings are disappointing, considering the theoretical context in which gifted students are educated in inclusive classrooms, and thus the regular class context is critical for the development of giftedness into talent (Gagné, 2004, 2021). On average, every teacher in Finland and Sweden encounters gifted students in mixed-ability classes on a daily basis. In this light, the frequency of acceleration should be considerably higher to meet gifted students’ needs effectively. It is also worth noting that, while both countries emphasise differentiated instruction (e.g., Finnish National Agency for Education, 2025; Paulsrud, 2022; Swedish National Agency for Education, 2024), differentiated instruction alone may not be sufficient. As Assouline et al. (2021) point out, the range of students’ needs and individual differences can be too broad for teachers to address, and they may lack the capacity required to provide accelerated content. A lack of resources and structures for gifted education may also constrain teachers’ ability to implement acceleration effectively. In the present study, almost 80% of respondents reported inadequate planning time for acceleration, and nearly 70% reported lacking access to acceleration materials. These high proportions likely reflect a system-level emphasis on low-achieving students while neglecting the needs of gifted students (e.g., Persson, 2010; Laine & Tirri, 2021).

Swedish teachers, compared with their Finnish counterparts, reported having more experience of teaching early-entrance, grade-skipping, and subject-accelerated students. Several factors could explain the observed differences. First, Swedish legislation allows early entrance to the preschool class (Education Act 800, 2010, Chapter 7, Section 11), an option that is not available to high-ability children in Finland; Laine et al. (2025) have recently suggested that Finland should consider allowing children to start pre-primary education a year earlier as well. Second, there is a notable difference in the diversity of the student population between the two countries. In 2022, the proportion of immigrant 15-year-old students was 7% in Finland and 21% in Sweden (Organisation for Economic Co-Operation and Development, 2023). In addition, the gap between socio-economically advantaged and disadvantaged students in mathematics achievement in PISA was considerably smaller in Finland than in Sweden (Organisation for Economic Co-Operation and Development, 2023). It is possible that such differences—for example, teaching large numbers of students with developing language skills in mixed-ability classrooms—are associated with Swedish teachers’ greater use of acceleration for their gifted students. Third, Finnish teachers may prioritise differentiated instruction over acceleration, as educational guidelines explicitly emphasise differentiated instruction as the foundation of all instruction (Finnish National Agency for Education, 2014, 2025). Finally, the Finnish participants frequently reported lacking information about their students’ prior acceleration: half did not know whether their students had started school early or skipped a grade, whereas the corresponding proportion in Sweden was only 13%. It is possible that, in Finland, the obligation to support gifted students and communicate about such support is not perceived as being as strong as the obligation regarding low-achieving students. Although gifted students should likewise be supported in ways that correspond to their level of ability, they are seldom mentioned in guiding educational documents, whereas support for low-achieving students and the documentation of such support is regulated in detail (Basic Education Act 628, 1998, Chapter 4; Finnish National Agency for Education, 2025). Nevertheless, it is important to note that even though Swedish teachers reported having taught accelerated students more often than their Finnish counterparts did, the numbers of such students they had encountered were low, reinforcing that acceleration in both countries is under-utilised as an educational strategy.

Several respondents reported that students’ early entrance or grade skipping had not affected their teaching. It is reasonable to assume that prior acceleration has been successful when teachers do not see notable differences between accelerated and other students in everyday classroom activities. In addition, a few teachers described the effect as positive. These results confirm prior evidence on the benefits of these forms of acceleration (e.g., Gagné & Gagnier, 2004; Hoogeveen et al., 2012; Rogers, 2015). Some teachers had also implemented special arrangements, such as offering guidance and more challenging tasks, to support the development of early-entrance and grade-skipping students. This finding demonstrates these teachers’ attentiveness to the needs of their gifted students, even when the students had already been subject to gifted education measures. The result is also in line with the notion by Assouline et al. (2021) that applying only one type of acceleration may be insufficient and that acceleration should not exclude the use of other gifted education options, such as differentiated instruction and enrichment.

Compared with early entrance and grade skipping, teachers were more aware of their students’ subject-based acceleration, presumably because they had more often been personally involved in such processes—for example, as subject teachers, homeroom teachers, or colleagues. Nevertheless, the reported number of subject-accelerated students was small, especially in Finland. Teachers applied various arrangements to support such students, including timetable adjustments, individual study materials, and collaboration with upper secondary schools. Subject-based acceleration also received positive mentions, which is consistent with its previously identified psychological (Ma, 2002; Rogers, 2015), social-emotional (Lee et al., 2010, 2012), and academic (Rogers, 2015) benefits.

No negative effects of grade skipping were reported. Some teachers had encountered student-related challenges with early entrance, and there were also a few negative experiences with subject acceleration. Neihart (2007) highlights that the positive socio-affective effects documented in the literature mainly reflect group-level results, whereas negative outcomes are seldom observed. However, at the individual level, negative effects are occasionally reported; these may relate to insufficient attention to students’ social readiness, emotional maturity, or motivation for acceleration (Neihart, 2007). Accordingly, Laine et al. (2025) emphasise that when educators select among different forms of acceleration, it is essential to consider the student’s overall situation and individual needs.

Regarding practices associated with curriculum compacting, the majority stated that they never or very rarely implemented diagnostic assessment to determine their gifted students’ skill level, nor did they engage them in independent and challenging project work. In contrast, they reported adjusting content a few times per month on average; more specifically, they eliminated curricular material on a monthly basis and, on average, bypassed content and set different assignments weekly. Almost one quarter reported instructing students to study higher-grade-level content at least monthly, whereas approximately the same proportion reported never doing so. Furthermore, one in ten teachers indicated that they applied individual teacher-defined forms of acceleration at least monthly, including additional instruction, challenging tasks, independent study, grouping, project work, and subject integration. It can thus be concluded that key features of curriculum compacting (Reis et al., 1998/2016; A. Robinson et al., 2007; VanTassel-Baska & Stambaugh, 2005) can be identified in how teachers applied acceleration, but usage appears unevenly distributed across teachers and practices. Nevertheless, the reported practices provide an excellent example of the various forms of acceleration that can be implemented in inclusive science education. Sharing these good examples is important to illustrate how teachers and schools can provide a supportive context in which talent can be realised (Gagné, 2004, 2021).

Finnish teachers expressed implementing versatile assessment practices more frequently than Swedish teachers did. This result may be explained by the strong emphasis on assessment in the latest curriculum reforms in Finland (Ketonen & Nieminen, 2024). In addition, there are no national examinations in basic education in Finland (Kupiainen et al., 2009), and teachers have broad autonomy in planning and conducting assessment (Ketonen & Nieminen, 2024). However, one may question whether assessment information in either country is being used adequately to inform educational personnel about students’ independent mastery level and the level at which they need support—known as the zone of proximal development (ZPD) (Vygotsky, 1978). Assessment tools need to have sufficiently high ceiling levels. Without such assessment evidence, educators can only guess at gifted (and other) students’ ZPD or potential readiness for acceleration. We cannot know from our survey whether assessment data enabled teachers to determine both students’ independent mastery level and the level at which they need support. However, our experience tells us that assessment tends to focus on expected-level achievement and that educational assessments often have a low ceiling level. This means that students might be identified as ‘above the expected level’, but little information may be available about how far above: are students one year ahead, three years ahead, or more?

The findings of this study—particularly the observed scarcity of acceleration—reinforce the argument of Assouline et al. (2021), according to which ‘access to acceleration is an issue of equity and fairness’ (p. 13). They further state that the key issue is not whether gifted students should be accelerated, but rather how many and what kinds of accelerative practices will benefit such students (Assouline et al., 2021). Equity can also be aligned to discussions about children’s rights: all students have the right to education and to learning (United Nations, n.d.), which are concepts different from simply being present at school. Advocacy regarding gifted students’ educational rights is linked to nine specific articles from the Convention on the Rights of the Child (United Nations, n.d.) in the work of Margrain et al. (2025). They argue that without attention to these rights, gifted children and students are unlikely to flourish. Examples include Article 3, the best interest of the child, and Article 5, appropriate guidance. In the specific subject areas of physics and chemistry, acceleration can be an important means of ensuring that gifted students can access information (Article 13) at levels aligned to their capability and learning potential (Article 29). Given these articulated rights (to which both Sweden and Finland are signatories), it is important to acknowledge that for some children their rights are not being met.

Some teachers also criticised the use of acceleration in physics and chemistry. They argued—in line with commonly presented counterarguments to acceleration (Assouline & Lupkowski-Shoplik, 2010; Ihrig & Degner, 2015)—that very few students are truly gifted in these subjects and that physics and chemistry are new to students in lower secondary school; hence, acceleration is not needed. Negative attitudes towards acceleration in general have been reported among Finnish primary-school teachers (Laine et al., 2019) and Swedish principals (Ivarsson, 2024). However, it is possible that science teachers’ negative attitudes are partly due to the nature of the natural sciences, rather than ‘general’ reluctance. As experimental (laboratory) work is an essential part of science instruction, it may be difficult for teachers to organise individual acceleration that includes hands-on science. Additionally, students may be less familiar with physics and chemistry than, for example, with mathematics, which may raise teachers’ concerns about students’ grasp of fundamental concepts and skills. A few teachers also spontaneously described their mathematics-related practices, likely because they had used acceleration more often in that subject. The findings highlight the need for future studies to examine acceleration—and attitudes towards it—in greater detail across different subjects.

One important finding is that teachers’ gender and length of teaching experience were not associated with the overall frequency of acceleration or with the use of curriculum-compacting practices. In contrast, teachers with knowledge of gifted education reported more frequent use of most practices than those without such knowledge. It is encouraging to observe a connection between teacher competence development and the implementation of acceleration. It is also worth noting that only half of the teachers reported possessing knowledge of gifted education, underscoring the need for increased training in acceleration and gifted education more broadly. Similar recommendations have been proposed by Laine and Tirri (2021), who advocate providing Finnish pre-service teachers with opportunities to experiment with diverse teaching practices across student groups. The need for pre-service and in-service training in adapting instruction for gifted students has also been acknowledged in Sweden (Westling Allodi, 2014).

What could be the next steps in increasing the use of acceleration in Nordic inclusive education? Heinbokel (2010) analysed the issue from a European perspective and proposed disseminating information on the benefits of acceleration, establishing parent associations, and promoting research on acceleration from diverse perspectives. The overall scarcity of acceleration documented in this study highlights a clear need in Finland and Sweden to communicate its benefits to teachers and teacher educators. In addition, it is essential to promote recognition of the equity perspective at the societal level to support the provision of accelerative options for gifted students. It would also be helpful to draw on the involvement of parents in promoting acceleration. In Sweden, there is ongoing association work around gifted education (Westling Allodi & Szabo, in press), whereas no active parental advocacy groups for gifted education exist in Finland (Makkonen et al., in press). In the Nordic context, much of the responsibility for gifted education lies with schools. Therefore, schools could—and should—regularly inform parents about gifted education opportunities (Laine et al., 2025), including options for acceleration. It should also be a priority to ensure that the structures of the education system facilitate the flow of information concerning gifted students; educators should be informed about their students’ prior acceleration. It could even be argued that such key transition information should be well-documented for all students’ optimal learning and psychological well-being. In addition, there is an obvious need for broader data collection regarding the number of accelerated students; no such information is recorded at the national level in either country. Regarding possibilities for further research, examining student and parent views on acceleration would complement the teacher perspective provided in this study. Reis et al. (1998/2016) also called for empirical studies on the achievement effects of curriculum compacting in secondary schools. As teachers in this study reported versatile ways of applying curriculum compacting, more detailed data on its use and (potential) benefits would be valuable. Moreover, as the CM scale used in the present study did not load cleanly and required item removal, future studies might consider adapting or validating a Nordic-specific instrument on curriculum compacting.

The findings may be limited in terms of content validity—that is, whether the instrument thoroughly covers the concept under study (Check & Schutt, 2012)—as we intentionally included only those practices considered relevant in the Nordic context. More specifically, we only addressed four practices, although at least 20 options have been identified in the literature (e.g., Southern & Jones, 2015). Due to the scarcity of suitable instruments, we compiled the survey from existing questionnaires and designed several items ourselves based on the literature. As we expected teachers to be unfamiliar with the concept of curriculum compacting, we used alternative approaches to examine its use. This procedure also entails limitations related to content validity. Although the questionnaire addressed several key practices associated with curriculum compacting, it is unlikely that it captured the full range of possible options. Nevertheless, the findings may contribute to the future development of instruments designed to investigate the implementation of curriculum compacting.

The mixed-methods design was considered beneficial, and the qualitative findings aligned with the quantitative ones. For example, many teachers reported not having observed any effects or challenges related to early entrance and grade skipping, and there were relatively few descriptions of special arrangements and teacher-defined forms of acceleration. These findings are consistent with the quantitative results indicating low acceleration implementation rates. Nevertheless, data triangulation beyond the survey format would have provided more robust conclusions. As mentioned earlier, we are continuing this research using interviews and lesson plans; however, such data are extensive and will therefore be addressed in a separate study.

It is also important to bear in mind the self-reported nature of the survey; participants may find it difficult to recall past details or the frequency of specific practices accurately. In addition, the possibility of self-serving bias (Forsyth, 2008) and social desirability bias (Paulhus, 2002) cannot be ruled out, although participants responded anonymously.

Caution is also warranted when generalising the results. Although the sample consists of teachers from different regions and with varying lengths of experience in both countries, a larger sample size would have been beneficial to establish whether the number of accelerated students increases in line with teaching experience. Additionally, the use of convenience sampling limits the generalisability; it is possible that teachers with a particular interest in gifted education were more likely to participate. Indeed, in the free-comment section, nine teachers spontaneously emphasised the importance of the topic, with one example being ‘This is a very good and important topic; hopefully, the development of the education for the gifted will become standard practice in Finland!’

## Figures and Tables

**Table 1:** Teachers’ experience of teaching early-entrance, grade-skipping, and subject-accelerated students.

Item	Sample	*n*	Agree	95% CI	Disagree	95% CI	No Such Information About Students	95% CI
Have you taught any students who have entered school earlier than enacted?	Total	158	59 (37%)	30−45	42 (27%)	20−34	57 (36%)	29−44
	Finland	96	24 (25%)	17−34	23 (24%)	16−33	49 (51%)	41−61
	Sweden	62	35 (57%)	44−68	19 (31%)	20−43	8 (13%)	6−23
Have you taught any students who have skipped grade(s)?	Total	156	41 (26%)	20−34	60 (39%)	31−46	55 (35%)	28−43
	Finland	94	12 (13%)	7−21	35 (37%)	28−47	47 (50%)	40−60
	Sweden	62	29 (47%)	35−59	25 (40%)	29−53	8 (13%)	6−23
Have you taught any students who have been accelerated using subject-based grade-level acceleration (e.g., they are in grade 7 but study grade 9 physics)?	Total	158	36 (23%)	17−30	97 (61%)	54−69	25 (16%)	11−22
	Finland	96	13 (14%)	8−21	61 (64%)	54−73	22 (23%)	15−32
	Sweden	62	23 (37%)	26−50	36 (58%)	46−70	3 (5%)	1−12

Note. Values indicate the number of teachers (*n*) with row percentages in parentheses.

**Table 2:** The number of early entrance, grade-skipping, and subject-accelerated students.

Practice	Sample	1 Student	2 Students	3 Students	4 Students	5 Students or More
Early entrance	Total	6 (4%)	9 (6%)	13 (8%)	1 (1%)	28 (18%)
	Finland	4 (4%)	4 (4%)	6 (6%)	0 (0%)	8 (8%)
	Sweden	2 (3%)	5 (8%)	7 (11%)	1 (2%)	20 (31%)
Grade-skipping	Total	14 (9%)	12 (8%)	6 (4%)	1 (1%)	7 (4%)
	Finland	7 (7%)	3 (3%)	0 (0%)	0 (0%)	1 (1%)
	Sweden	7 (11%)	9 (14%)	6 (9%)	1 (2%)	6 (9%)
Subject-based acceleration	Total	15 (9%)	8 (5%)	5 (3%)	2 (1%)	6 (4%)
	Finland	8 (8%)	4 (4%)	0 (0%)	1 (1%)	0 (0%)
	Sweden	7 (11%)	4 (6%)	5 (8%)	1 (2%)	6 (9%)

Note. Percentages are based on the full samples (*N* = 160, *n*_FI_ = 96, *n*_SE_ = 64).

**Table 3:** Individual items related to curriculum compacting.

Item	*M* ^a^	*SD*	Never	Very Rarely	About Once a Semester	About Once a Month	About Once a Week	A Few Times a Week	Every Day
Main items									
I have used diagnostic assessment to determine the knowledge and skill level of my gifted students.	2.17 ^b^	1.20	55 (36%)	47 (31%)	33 (21%)	13 (8%)	3 (2%)	2 (1%)	1 (1%)
I have instructed a gifted student to study content from a higher grade level.	2.50 ^c^	1.44	42 (27%)	58 (37%)	18 (12%)	21 (13%)	9 (6%)	7 (4%)	1 (1%)
I have instructed a gifted student to work independently on a challenging project during their school hours.	2.27 ^d^	1.24	48 (31%)	53 (34%)	32 (21%)	12 (8%)	7 (5%)	2 (1%)	1 (1%)
I have instructed a gifted student to work independently on a challenging project during their free time.	1.90 ^c^	1.08	72 (46%)	47 (30%)	22 (14%)	12 (8%)	1 (1%)	2 (1%)	0 (0%)
Clarifying items									
I have instructed a gifted student to study content from a higher grade level as part of their ordinary instruction in their own class/group.	3.04 ^e^	1.48	10 (9%)	44 (39%)	25 (22%)	15 (13%)	9 (8%)	9 (8%)	2 (2%)
I have instructed a gifted student to study content from a higher grade level by attending higher-grade-level classes for part of the time.	1.41 ^e^	0.96	85 (75%)	21 (18%)	4 (4%)	1 (1%)	1 (1%)	1 (1%)	1 (1%)
I have instructed a gifted student to study content from a higher grade level by attending higher-grade-level classes full-time.	1.29 ^f^	0.86	94 (83%)	13 (12%)	3 (3%)	0 (0%)	2 (2%)	0 (0%)	1 (1%)
I have instructed a gifted student to study content from a higher grade level by working independently (e.g., at home or in another out-of-school setting).	2.38 ^e^	1.31	28 (25%)	49 (43%)	16 (14%)	14 (12%)	3 (3%)	2 (2%)	2 (2%)

Note. ^a^ On a Likert-type scale ranging from 1 to 7; higher values indicate frequent use. ^b^ *n* = 154, ^c^ *n* = 156, ^d^ *n* = 155, ^e^ *n* = 114, ^f^ *n* = 113. Percentages are based on respondent counts; see superscripts.

**Table 4:** Items, means, standard deviations, Cronbach’s alphas, principal component loadings (Comp. 1 & Comp. 2), communalities (*h*^2^), and percentages of variance.

Scale/Item	*α*	*M* ^a^	95% CI	*SD*	Comp. 1	Comp. 2	*h* ^2^
Assessment	0.827	3.19 ^b^	3.01−3.37	1.09			
I use a variety of assessment formats.		3.03 ^c^	2.83−3.22	1.22	0.89	0.02	0.80
I use ongoing assessment strategies.		3.27 ^d^	3.07−3.47	1.25	0.87	0.00	0.76
I use a balanced assessment system.		3.25 ^e^	3.04−3.46	1.27	0.81	−0.01	0.65
Content adjustment	0.702	2.18 ^f^	2.02−2.34	1.03			
I eliminate curricular material students have already mastered.		1.51 ^g^	1.33−1.70	1.16	−0.16	0.87	0.71
I allow students to bypass content that they have already mastered.		2.37 ^g^	2.13−2.61	1.52	0.03	0.83	0.70
I give different assignments for students who have mastered regular material.		2.63 ^g^	2.44−2.83	1.21	0.22	0.66	0.56
Percent of variance					44.30	25.11	

Note. ^a^ On a Likert-type scale ranging from 0 to 5; higher values indicate frequent use. ^b^ *n* = 142, ^c^ *n* = 151, ^d^ *n* = 154, ^e^ *n* = 145, ^f^ *n* = 155, ^g^ *n* = 156.

**Table 5:** Spearman’s rho correlations between the CM scales and the background variables.

Variable	Assessment	Content Adjustment	Country	Gender	Knowledge of Gifted Education	Teaching Experience
Assessment	–					
Content adjustment	0.25 **	–				
Country	−0.37 **	0.10	–			
Gender	−0.01	−0.03	−0.04	–		
Knowledge of gifted education	−0.23 **	−0.27 **	0.00	0.02	–	
Teaching experience	−0.06	0.14	0.16	−0.02	0.01	–

** *p* < 0.01.

## Data Availability

The datasets presented in this article are not readily available because the data are part of an ongoing study. Requests to access the datasets should be directed to the corresponding author.

## Abbreviations

The following abbreviations are used in this manuscript:

CM	Curriculum Modifications
CPS-R	Classroom Practices Survey–Revised
FI	Finland
PCA	Principal component analysis
PISA	Programme for International Student Assessment
SE	Sweden
STEM	Science, technology, engineering, and mathematics
ZPD	Zone of proximal development

## Appendix A. The Survey Questionnaire

Introduction, ethical section, and background questions (not reproduced here).

Acceleration

In this section, we use the concept of acceleration, which means enabling a student to progress at a faster pace than usual (=faster than most other students).

1. Have you taught any student(s) who has/have entered school earlier than enacted (e.g., started school one year earlier)? Yes/No/I do not have such information about my students
  How many such students have you taught? 1, 2, 3, 4, 5 or more
  How has this acceleration influenced your teaching practices concerning such student(s)?
2. Have you taught any student(s) who has/have been grade-level accelerated (whole year or several years), in other words, who have skipped grade(s)? Yes/No/I do not have such information about my students
  How many such students have you taught? 1, 2, 3, 4, 5 or more
  How has this acceleration influenced your teaching practices concerning such student(s)?
3. Have you taught any student(s) who has/have been accelerated using subject-specific grade-level acceleration (e.g., they are in grade 7 but study grade 9 physics)? Yes/No/I do not have such information about my students
  How many such students have you taught? 1, 2, 3, 4, 5 or more
  How has this acceleration influenced your teaching practices concerning such student(s)?
4. Please assess the frequency of the following practices using the scale: Never, Very rarely, About once in semester, About once a month, About once a week, A few times a week, Every day
  As a teacher, I have…
  used diagnostic assessment to determine the knowledge and skill level of my gifted students
  instructed a gifted student to study content from a higher grade level
  instructed a gifted student to work independently on a challenging project during their school hours
  instructed a gifted student to work independently on a challenging project during their free time
  implemented other type(s) of teaching arrangements to accelerate
  Please specify what other type(s) of teaching arrangements.
  4.1. I have instructed a gifted student to study content from a higher grade level…
    as part of their ordinary instruction in their own class/group
    by attending higher-grade-level classes for part of the time
    by attending higher-grade-level classes full-time
    by working independently (e.g., at home or in another out-of-school setting)
    Scale: Never, Very rarely, About once in semester, About once a month, About once a week, A few times a week, Every day
5. On average, I use acceleration for a gifted student (choose one): Never, Very rarely, In at least 25% of the lessons, In at least 50% of the lessons, In at least 75% of the lessons, In almost every lesson, In every lesson
6. Curriculum modifications for gifted students
  Please reflect on your own actions and indicate how often the following statements apply to you: Never, Once a month, or less frequently, A few times a month, A few times a week, Daily, More than once a day
  - I use pretests to determine mastery.
  - I eliminate curricular material students have already mastered.
  - I give different assignments for students who have mastered regular material.
  - I assign different homework based on achievement level.
  - I use ongoing assessment strategies.
  - I use a variety of assessment formats.
  - I use culturally responsive curricula to engage all students.
  - I use a balanced assessment system.
  - I use technology to differentiate instruction.
  - I allow students to bypass content that they have already mastered.
  - I use tiered lesson plans.
7. Resources at school
  Please select the option that you think best reflects the resources available to you to support your gifted students: Strongly disagree, Disagree, I don’t know, Agree, Strongly agree
  As a teacher, I have…
  adequate planning time to accelerate instruction.
  access to the instructional materials necessary to accelerate instruction.

## Appendix B

**Table A1:** Cross-tabulation of teaching experience and the number of early-entrance students.

Number of Students\Teaching Experience	≤10 Years	11–20 Years	≥21 Years	Total
1–3 students	13 (72%)	5 (36%)	10 (40%)	28 (49%)
≥4 students	5 (28%)	9 (64%)	15 (60%)	29 (51%)
Total	18 (100%)	14 (100%)	25 (100%)	57(100%)

Note. Values indicate the number of teachers (*n*) with column percentages in parentheses.

**Table A2:** Cross-tabulation of teaching experience and the number of grade-skipping students.

Number of Students\Teaching Experience	≤10 Years	11–20 Years	≥21 Years	Total
1–3 students	12 (100%)	6 (60%)	14 (78%)	32 (80%)
≥4 students	0 (0%)	4 (40%)	4 (22%)	8 (20%)
Total	12 (100%)	10 (100%)	18 (100%)	40 (100%)

Note. Values indicate the number of teachers (*n*) with column percentages in parentheses.

**Table A3:** Cross-tabulation of teaching experience and the number of subject-accelerated students.

Number of Students\Teaching Experience	≤10 Years	11–20 Years	≥21 Years	Total
1–3 students	9 (90%)	9 (75%)	10 (71%)	28 (78%)
≥4 students	1 (10%)	3 (25%)	4 (29%)	8 (22%)
Total	10 (100%)	12 (100%)	14 (100%)	36 (100%)

Note. Values indicate the number of teachers (*n*) with column percentages in parentheses.

**Table A4:** Country comparison: Individual items related to curriculum compacting.

Item	Finland			Sweden			*U*	*Z*	*p*	*r*
	*Md*	*IQR*	*MR*	*Md*	*IQR*	*MR*				
Main items										
I have used diagnostic assessment to determine the knowledge and skill level of my gifted students.	2 ^a^	2	68.89	2 ^b^	2	90.63	3637.5	3.092	0.002	0.25
I have instructed a gifted student to study content from a higher grade level.	2 ^c^	2	71.85	2 ^d^	2	88.58	3539.0	2.354	0.019	0.19
I have instructed a gifted student to work independently on a challenging project during their school hours.	2 ^a^	2	68.28	2 ^d^	1	92.58	3787.0	3.441	<0.001	0.28
I have instructed a gifted student to work independently on a challenging project during their free time.	2 ^c^	1	75.31	2 ^d^	2	83.34	3214.0	1.164	0.244	0.09
Clarifying items										
I have instructed a gifted student to study content from a higher grade level as part of their ordinary instruction in their own class/group.	2 ^e^	1	49.92	3 ^f^	3	67.55	2085.0	2.926	0.003	0.27
I have instructed a gifted student to study content from a higher grade level by attending higher-grade-level classes for part of the time.	1 ^e^	0	52.08	1 ^f^	1	64.69	1945.0	2.651	0.008	0.25
I have instructed a gifted student to study content from a higher grade level by attending higher-grade-level classes full-time.	1 ^e^	0	53.03	1 ^g^	1	62.38	1818.0	2.305	0.021	0.22
I have instructed a gifted student to study content from a higher grade level by working independently (e.g., at home or in another out-of-school setting).	2 ^e^	1	56.62	2 ^f^	2	58.76	1650.0	0.347	0.729	0.03

Note. ^a^ *n* = 93, ^b^ *n* = 61, ^c^ *n* = 94, ^d^ *n* = 62, ^e^ *n* = 65, ^f^ *n* = 49, ^g^ *n* = 48.

**Table A5:** Knowledge of gifted education: Individual items related to curriculum compacting.

Item	Knowledge of Gifted Education			No Knowledge of Gifted Education			*U*	*Z*	*p*	*r*
	*Md*	*IQR*	*MR*	*Md*	*IQR*	*MR*				
Main items										
I have used diagnostic assessment to determine the knowledge and skill level of my gifted students.	2 ^a^	2	82.40	2 ^b^	1	65.01	2059.5	−2.586	0.010	0.21
I have instructed a gifted student to study content from a higher grade level.	2 ^c^	2	85.30	2 ^d^	2	63.99	1979.0	−3.128	0.002	0.26
I have instructed a gifted student to work independently on a challenging project during their school hours.	2 ^c^	2	81.64	2 ^d^	2	67.90	2261.0	−2.020	0.043	0.17
I have instructed a gifted student to work independently on a challenging project during their free time.	2 ^c^	2	79.54	2 ^d^	1	70.15	2422.5	−1.421	0.155	0.12
Clarifying items										
I have instructed a gifted student to study content from a higher grade level as part of their ordinary instruction in their own class/group.	3 ^e^	2	55.28	3 ^f^	2	55.82	1477.0	.091	0.927	0.01
I have instructed a gifted student to study content from a higher grade level by attending higher-grade-level classes for part of the time.	1 ^e^	1	60.12	1 ^f^	0	48.83	1162.5	−2.395	0.017	0.23
I have instructed a gifted student to study content from a higher grade level by attending higher-grade-level classes full-time.	1 ^e^	0	56.86	1 ^g^	0	52.25	1309.0	−1.158	0.247	0.11
I have instructed a gifted student to study content from a higher grade level by working independently (e.g., at home or in another out-of-school setting).	2 ^e^	2	60.77	2 ^f^	2	47.89	1120.0	−2.188	0.029	0.21

Note. ^a^ *n* = 76, ^b^ *n* = 71, ^c^ *n* = 77, ^d^ *n* = 72, ^e^ *n* = 65, ^f^ *n* = 45, ^g^ *n* = 44.
